# Multi-drug resistant bacteria isolates from lymphatic filariasis patients in the Ahanta West District, Ghana

**DOI:** 10.1186/s12866-022-02624-9

**Published:** 2022-10-11

**Authors:** Bill Clinton Aglomasa, Cynthia Kyerewaa Adu-Asiamah, Samuel Opoku Asiedu, Priscilla Kini, Emmanuel Kobla Atsu Amewu, Kennedy Gyau Boahen, Solomon Wireko, Isaac Kingsley Amponsah, Yaw Duah Boakye, Vivian Etsiapa Boamah, Alexander Kwarteng

**Affiliations:** 1 Department of Pharmaceutics, College of Health Science, Kwame Nkrumah University of Science and Technology, Kumasi, Ghana; 2 Kumasi Centre for Collaborative Research in Tropical Medicine, Kwame Nkrumah University of Science and Technology, Kumasi, Ghana; 3 Department of Clinical Microbiology, School of Medical Sciences, Kwame Nkrumah University of Science and Technology, Kumasi, Ghana; 4 Department of Biochemistry and Biotechnology, College of Science, Kwame Nkrumah University of Science and Technology, Kumasi, Ghana; 5 Department of Laboratory Technology, Kumasi Technical University, Kumasi, Ghana; 6 Department of Pharmacognosy, College of Health Science, Kwame Nkrumah University of Science and Technology, Kumasi, Ghana

**Keywords:** Lymphatic filariasis, Antimicrobial resistance, Multi-drug resistance, Methicillin-resistant *Staphylococcus aureus*, Extended-spectrum beta-lactamase, Secondary bacterial infection, Acute dermatolymphangioadenitis

## Abstract

**Background:**

Antimicrobial resistance is associated with increased morbidity in secondary infections and is a global threat owning to the ubiquitous nature of resistance genes in the environment. Recent estimate put the deaths associated with bacterial antimicrobial resistance in 2019 at 4.95 million worldwide. Lymphatic filariasis (LF), a Neglected Tropical Disease (NTD), is associated with the poor living in the tropical regions of the world. LF patients are prone to developing acute dermatolymphangioadenitis (ADLA), a condition that puts them at risk of developing secondary bacterial infections due to skin peeling. ADLA particularly worsens the prognosis of patients leading to usage of antibiotics as a therapeutic intervention. This may result in inappropriate usage of antibiotics due to self-medication and non-compliance; exacerbating antimicrobial resistance in LF patients. In this perspective, we assessed the possibilities of antimicrobial resistance in LF patients. We focused on antibiotic usage, antibiotic resistance in *Staphylococcus aureus*, *Escherichia coli* and *Pseudomonas aeruginosa* isolates and looked at genes (*mec*A and Extended-spectrum beta-lactamase [*bla*CTX-M, *bla*SHV and *bla*TEM]) coding for resistance in multi-drug resistant (MDR) bacterial isolates.

**Results:**

Of the sixty (60) participants, fifty-four (*n* = 54, 90%) were within 31–60 years of age, twenty (*n* = 20, 33.33%) were unemployed and thirty-eight (*n* = 38, 50.67%) had wounds aged (in months) seven (7) months and above. Amoxicillin (54%) and chloramphenicol (22%) were the most frequently used antibiotics for self-medication. *Staphylococcus aureus* isolates (*n* = 26) were mostly resistant to penicillin (*n* = 23, 88.46%) and least resistant to erythromycin (*n* = 2, 7.69%). *Escherichia coli* isolates (*n* = 5) were resistant to tetracycline (*n* = 5, 100%) and ampicillin (*n* = 5, 100%) but were sensitive to meropenem (*n* = 5, 100%). *Pseudomonas aeruginosa* isolates (*n* = 8) were most resistant to meropenem (*n* = 3, 37.50%) and to a lesser ciprofloxacin (*n* = 2, 25%), gentamicin (*n* = 2, 25%) and ceftazidime (*n* = 2, 25%). Multi-drug resistant methicillin resistant *Staphylococcus aureus* (MRSA), cephalosporin resistant *Escherichia coli.* and carbapenem resistant *Pseudomonas aeruginosa* were four (*n* = 4, 15.38%), two (*n* = 2, 40%) and two (*n* = 2, 25%) respectively. ESBL (*bla*CTX-M) and *mec*A genes were implicated in the resistance mechanism of *Escherichia coli* and MRSA, respectively.

**Conclusion:**

The findings show presence of MDR isolates from LF patients presenting with chronic wounds; thus, the need to prioritize resistance of MDR bacteria into treatment strategies optimizing morbidity management protocols. This could guide antibiotic selection for treating LF patients presenting with ADLA.

**Supplementary Information:**

The online version contains supplementary material available at 10.1186/s12866-022-02624-9.

## Background

Lymphatic filariasis (LF), a neglected tropical disease (NTD), is as a result of *Wuchereria bancrofti*, *Brugia malayi* or *Brugia timori,* transmitted through the bites of mosquitoes (from the genera Culex, Anopheles, Mansonia and Aedes) [1]. Since the inception of the Global Programme to Eliminate Lymphatic Filariasis (GPELF), efforts have been made at eliminating LF through mass drug administration (MDA) and morbidity management and disability prevention [MMDP] [2]. This has seen the likes of Palau meeting the criteria for eliminating LF as a public health concern [3]. Although this is welcoming, it falls short of the schedule of 2020 being the year to eradicate LF or be in the post MDA stage [4]. MMDP continues to remain one of the measures of managing LF patients including the complications that come with acute dermatolymphangioadenitis (ADLA). In the progression of LF, ADLA results in fever, chills, malaise, diffuse inflammation, swelling of the limbs, lymphangitis, adenitis and eventually, skin peeling [5]. This exposes LF patients to secondary bacterial infections which worsen their prognosis through increased morbidity and mortality [6–11].

Secondary infections occur during or after an infection of a primary pathogen [6]. In recent years, pathogenic bacteria have become problematic due to increasing antimicrobial resistance (AMR) [12]. Bacterial infections associated with other diseases are four times more likely to result in the death of a patient compared with bacterial infections alone [13]. *Escherichia coli (E. coli)*, *Staphylococcus aureus (S. aureus)* and *Haemophilus influenza* remain some of the commonest bacteria isolates implicated in secondary infections [14]. While these have been mainly due to nosocomial infections, the resurgence of the One Health approach is giving evidence of community infections as well [15, 16]. Some *S. aureus* found in hospital and community environments are known to be penicillinase producers [17]. *E. coli* is located in different niches aside its intestinal habitat [18]. *Pseudomonas aeruginosa (P. aeruginosa)* is second to *E. coli* in terms of being the most significant MDR Gram-negative pathogen [19]. Virulence in *S. aureus*, *E. coli* and *P. aeruginosa* has thus been potentiated by mobile genetic elements and the innate resistances they possess [20]. Co-infections with these agents could result in abscess formation and necrotizing infections in LF patients [21–23].

Bacterial resistance may occur due to transient changes in bacterial permeability to antibiotics, drug indifference, biofilm formation or presence of persistent cells [24]. Acquired resistance has received attention when it comes to resistance in *S. aureus*, *E. coil* and *P. aeruginosa* bacteria strains due to their virulence [25–29]. Among these are *mec*A and ESBL genes. The *mec*A gene codes for resistance in *S. aureus* by producing penicillin-binding proteins (PBPs) that have low affinity for beta-lactam antibiotics. The *mec*A gene evolution is associated with anthropogenic activities that resulted in a core gene in *Staphylococcus sciuri* becoming a resistance determinant for broad spectrum beta-lactams in *S. aureus* [30]. Extended-spectrum beta-lactamases (ESBLs) are a group of enzymes known for their ability to hydrolyse ESBL antibiotics and in are inhibited by beta-lactamase inhibitors [31]. ESBLs are known to have evolved from point mutations in the beta-lactamase (*bla*) encoding genes [32]. Among the many families of ESBLs are *bla*TEM, *bla*SHV and* bla*CTX-M genes with the former being the predominant ESBL currently [32–34].

Aside the virulent nature of these organisms and genes, selective pressure due to antimicrobial usage has evolved a cocktail of resistance mechanisms that evade current antibiotics including penicillin, cephalosporins and carbapenem [35, 36]. Here, we applied a cross-sectional study and systematically collected bacteria isolates from eight endemic communities in the Ahanta West district, Ghana. MDR *S. aureus*, *E. coli* and *P. aeruginosa* were isolated from LF patients presenting with wounds. We assessed participants antibiotic usage, screened isolates resistance to commonly used antibiotics and presence of *mec*A, *bla*SHV, *bla*TEM and *bla*CTX-M genes. We found evidence of self-medication as a possible determinant of the antimicrobial resistance seen in the bacteria isolates. Our analysis informs the usage of antibiotics and MDR patterns among LF patients.

## Results

### Demography and wound characteristics of patients

Sixty (60) individuals were recruited for the study with the female sex having the highest representation (*n* = 42, 70%). Other demographic data on participants including age, employment status and wound characteristics (cause of wound, dimension, age (month) of wound existence and signs/symptoms of infections) were also captured (Table 1). Majority (*n* = 53, 70.67%) of the wounds participants presented were caused by the underlying condition of LF while fifteen (*n* = 15, 20%) were due to trauma suffered by the patients.

**Table 1:** Sociodemography of participants

**Variables**	**N**	**%**	**SD**	**Mean**	**Variables**	**N**	**%**	**Variables**	**N**	**%**
**Age**					**Dimension of wound**			**Appearance of wound**		
18–30	6	10.00	2.53	21.00	0–5 cm	39	52.00	Moist	41	54.67
31–40	18	30.00	2.48	35.39	6–10 cm	7	9.33	Dry	34	45.33
41–50	20	33.33	2.64	45.30	> 15 cm	29	38.67			
51–60	16	26.67	3.24	54.88				**Participants with signs/symptoms of wounds (*N* = 55)**		
	60	100.00	10.67	42.45						
					**Age of wound (month)**			**Pain**		
**Sex**					< 1	13	17.33	Yes	38	69.09
Male	18	30.00			1–2	14	18.67	No	17	30.91
Female	42	70.00			3–6	10	13.33			
					> 7	38	50.67	**Swelling**		
								Yes	30	54.55
**Marital status**					**Signs of infection**			No	25	45.45
Single	50	83.33			Yes	55	73.33			
Married	10	16.67			No	20	26.67	**Pus**		
								Yes	28	50.91
**Employment status**					**Leg staging**			No	27	49.09
Farming	22	36.67			I	2	3.33			
Fishing	18	30.00			II	10	16.67	**Redness**		
Unemployed	20	33.33			III	22	36.67	Yes	26	47.27
					IV	9	15.00	No	29	52.73
**Cause of wound**					V	8	13.33			
Underlying condition	53	70.67			VI	8	13.33	**Smell**		
Trauma	15	20.00			VII	1	1.67	Yes	29	52.73
Unknown	7	9.33						No	26	47.27

Key: *SD* Standard deviation, *M* Mean, *N* Number of participants, *%* Percentage

### Antibiotics usage in participants

To gain insight on commonly used antibiotic in the study communities, participants were asked to respond to whether or not they use antibiotics and to name the antibiotic (if they had used one in the last three months) (Table 2). The antibiotics commonly used were amoxicillin, chloramphenicol, ampicillin, tetracycline, penicillin, flucloxacillin and clindamycin. Majority of the participants taking antibiotics (*n* = 36, 72%) were on beta-lactam antibiotics with the rest (*n* = 14, 28%) on chloramphenicol, tetracycline and lincosamide.

**Table 2:** Antibiotic (class) used by participants who knew the antibiotic they use

Antibiotic class	Antibiotic	Number of participants (%)
Beta-lactam	Amoxicillin	27 (54)
	Ampicillin	5 (10)
	Penicillin	2 (4)
	Flucloxacillin	2 (4)
Phenicols	Chloramphenicol	11 (22)
Tetracycline	Tetracycline	2 (4)
Lincosamide	Clindamycin	1 (2)
Total		
4	7	50 (100%)

### Resistance pattern of all isolates

A total of seventy-five (75) wound swabs were collected during the study. Participants with multiple wounds were sampled with different swabs to prevent cross contamination. From the swabs, a total of thirty-nine (39) isolates were obtained; comprising *E. coli* (*n* = 5), *P. aeruginosa* (*n* = 8) and *S. aureus* (*n* = 26) (Table 3). All *E. coli* strains were resistant to sulfamethoxazole-trimethoprim, ampicillin, tetracycline and ampicillin-sulbactam. Sensitivity tests for *P. aeruginosa* were performed but not reported for sulfamethoxazole-trimethoprim, ampicillin, tetracycline, ampicillin-sulbactam, chloramphenicol, ceftriaxone and cefuroxime as there are no disk and minimum inhibitory concentration (MIC) breakpoints in European Committee on Antimicrobial Susceptibility Testing (EUCAST) [61] and Clinical & Laboratory Standards Institute (CLSI) protocols (Table 4). However, it is recommended they are reported as resistant when these antibiotics are tested although a small proportion of *P. aeruginosa* isolates are known to be sensitive to them. The *S. aureus* isolates were mostly resistant to penicillin (*n* = 23, 88.46%) and least resistant to erythromycin (*n* = 2, 7.69%). Eleven (11) isolates were MDR (resistant to three or more classes of antibiotics) (Table 4).

**Table 3:** Resistance pattern of *S. aureus*, *E. coli* and *P. aeruginosa* in LF patients

Isolates (n)	Resistance frequency of bacteria to antibiotics n (%)										
**Gram-positive**	**TET**	**CIP**	**CN**	**SXT**	**CC**	**FOX**	**C**	**E**	**P**		
*S. aureus* (26)	15(57.69)	3(11.54)	3(11.54)	4(15.38)	5(19.23)	5(19.23)	20(76.92)	2(7.69)	23(88.46)		
**Gram-negative**	**TET**	**CIP**	**CN**	**SXT**	**C**	**MEM**	**AM**	**SAM**	**CAZ**	**CRO**	**CXM**
*E. coli* (5)	5(100.00)	3(60.00)	2(40.00)	5(100.00)	2(40.00)	0(0.00)	5(100.00)	5(100.00)	3(60.00)	3(60.00)	3(60.00)
*P. aeruginosa* (8)	ND	2(25.00)	2(25.00)	ND	ND	3(37.50)	ND	ND	2(25.00)	ND	ND

Key: *ND* Not determined as there are no breakpoints in EUCAST/CLSI protocols, *SXT* Sulfamethoxazole-trimethoprim, *TET *Tetracycline, *P* Penicillin, *CN* Gentamicin, *SAM* Ampicillin-sulbactam, *CRO* Ceftriaxone, *FOX* Cefoxitin, *CXM* Cefuroxime, *C* Chloramphenicol, *CIP* Ciprofloxacin, *CC* Clindamycin, *E* Erythromycin, *MEM* Meropenem, *AM* Ampicillin, *CAZ* Ceftazidime

**Table 4:** Distribution of MDR *S. aureus*, *E. coli* and *P. aeruginosa* in LF patients

Organism	Total isolates	Isolates confirmed as MDR	Percentage of MDR (%)
***E. coli***	5	2	40.00
***P. aeruginosa***	8	5	62.50
***S. aureus***	26	4	15.38
Total	39	11	28.20

### Distribution of MDR MRSA, *E. coli* and *P. aeruginosa* in LF patients

The eleven (11) MDR isolates comprised four (*n* = 4, 36.36%) MRSA, two (*n* = 2, 18.18%) *E. coli* and five (*n* = 5, 45.45%) *P. aeruginosa* (Table 4). Of the twenty-six (26) *S. aureus* isolates, four (*n* = 4, 15.38%) were resistant to cefoxitin and one antibiotic in 2 or more classes (Tables 3 and 4). Respectively, the 11 multi-drug resistant isolates were made up of 15.38, 40.00 and 62.50% of MRSA, *E. coli,* and *P. aeruginosa* (Table 4).

### Sensitivity profile of MDR isolates in LF

#### Sensitivity profile of MDR MRSA

**Fig. 1:**
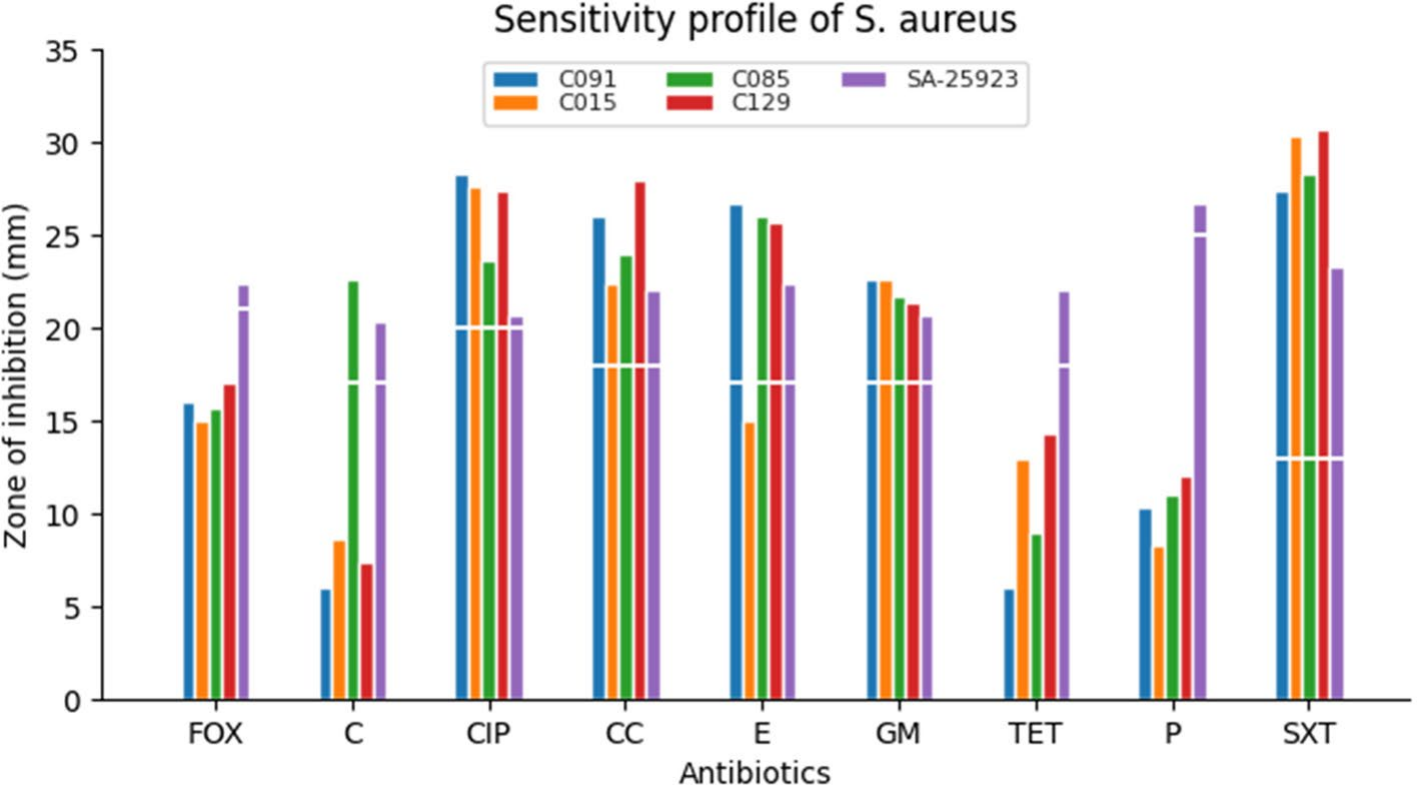
Sensitivity profile of MRSA against different antibiotics Key: *SXT* Sulfamethoxazole-trimethoprim, *FOX* Cefoxitin, *C* Chloramphenicol, *CIP* Ciprofloxacin, *CC* Clindamycin, *E* Erythromycin, *CN* Gentamicin, *TET* Tetracycline, *P* Penicillin. The breaks (white line) in the graph indicate resistant isolates (where the bars are below the break) or sensitive isolates (bars extend beyond the break) to the respective antibiotics listed

The MRSA isolates were all sensitive to sulfamethoxazole-trimethoprim, clindamycin, gentamicin and ciprofloxacin. All the MRSA isolates were resistant to cefoxitin, tetracycline and penicillin (Fig. 1). The American Type Culture Collection [ATCC] 25923 *S. aureus* strain was used as control.

#### Sensitivity profile of *E. coli*

**Fig. 2:**
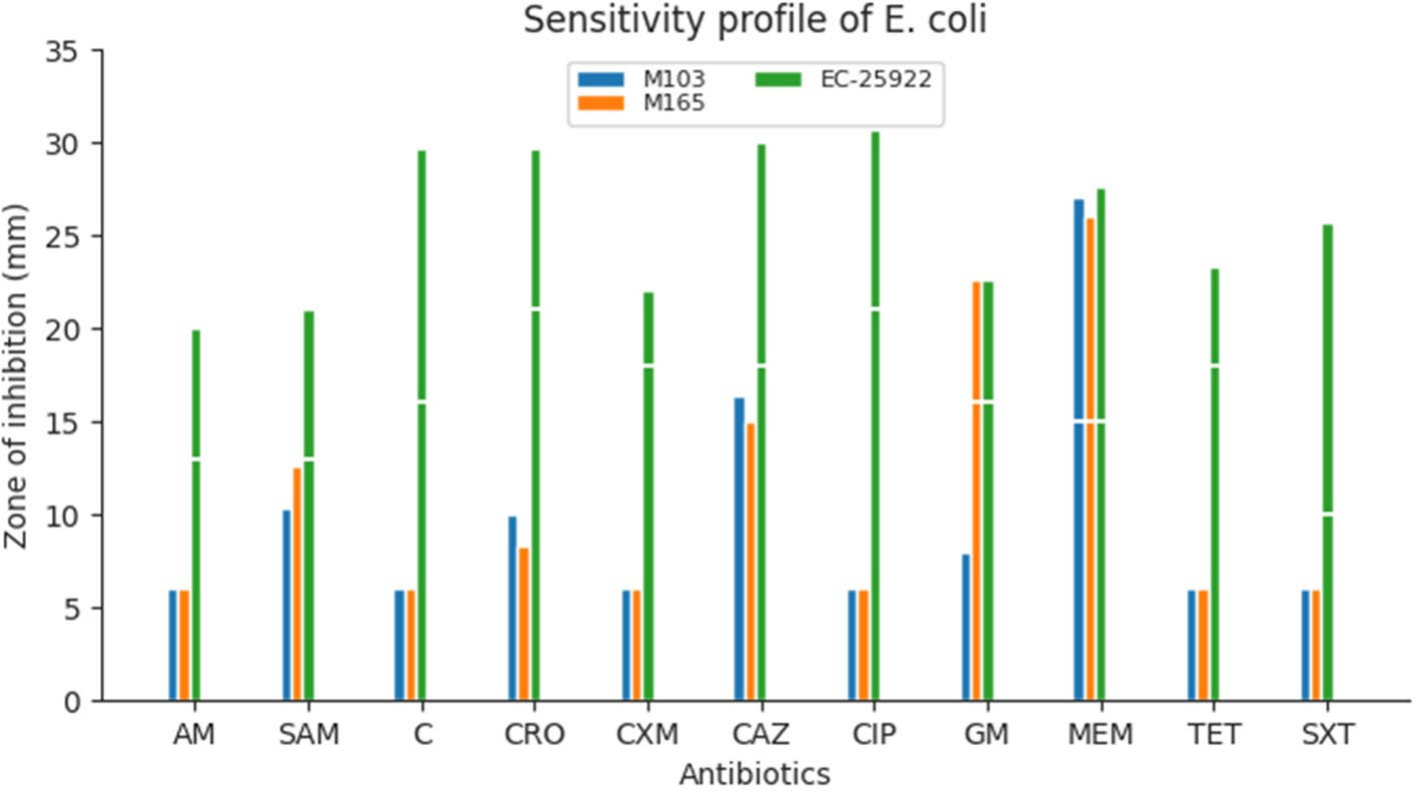
Sensitivity profile of *E. coli* against different antibiotics Key: *SXT* Sulfamethoxazole-trimethoprim, *TET* Tetracycline, *AM* Ampicillin, *SAM* Ampicillin-sulbactam, *C* Chloramphenicol, *CRO* Ceftriaxone, *CXM* Cefuroxime, *CAZ* Ceftazidime, *CIP* Ciprofloxacin, *GM* Gentamicin, *MEM* Meropenem. The breaks (white line) in the graph indicate resistant isolates (where the bars are below the break) or sensitive isolates (bars extend beyond the break) to the respective antibiotics listed

MDR *E. coli* isolates were resistant to all antibiotics, including the third generation cephalosporins (cefuro-xime, ceftazidime and ceftriaxone), except meropenem. One isolate was also susceptible to gentamicin (Fig. 2). The ATCC 25922 strain was used as control.

#### Sensitivity profile of *P. aeruginosa*

**Fig. 3:**
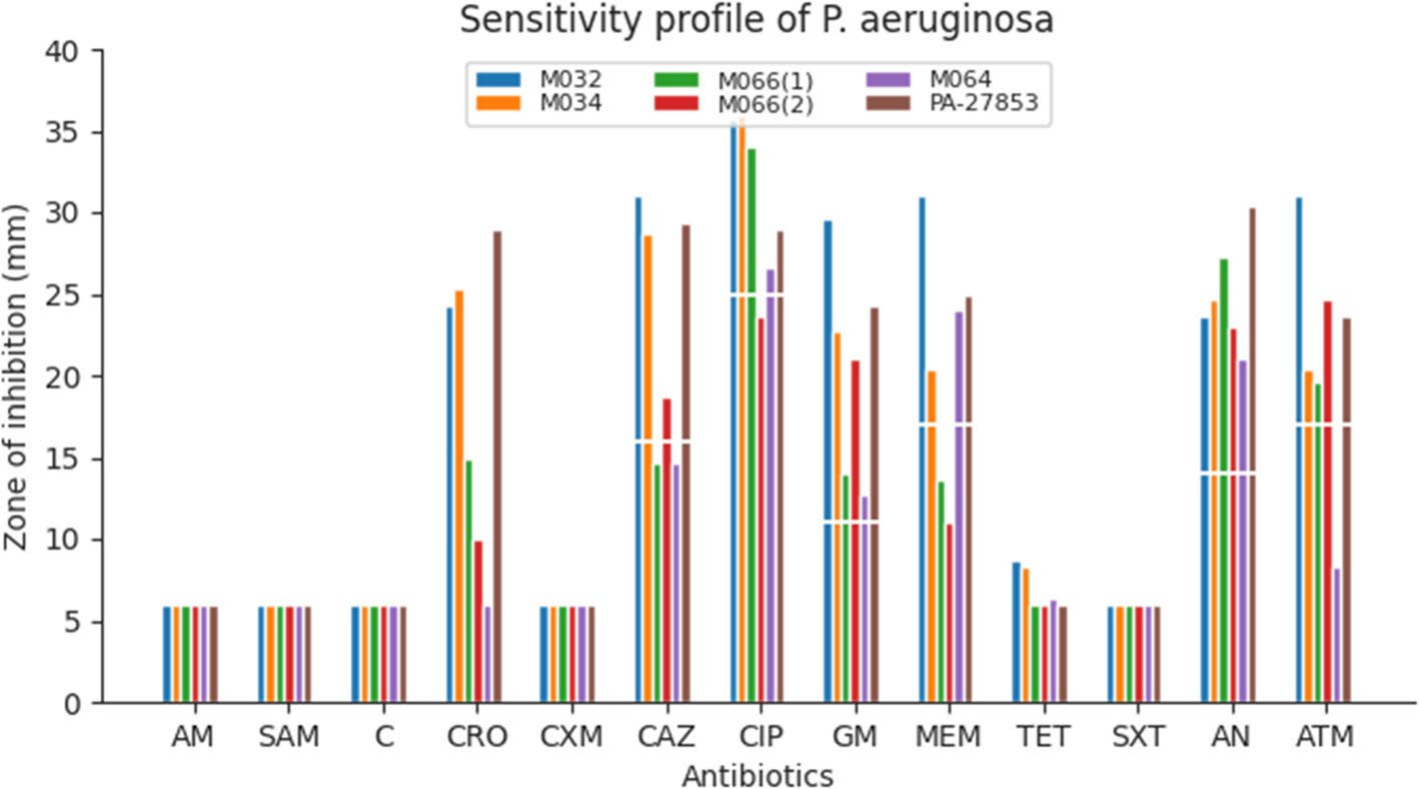
Sensitivity profile of *P. aeruginosa* against different antibiotics Key: *SXT* Sulfamethoxazole-trimethoprim, *AM *Ampicillin, *SAM *Ampicillin-sulbactam, *C* Chloramphenicol, *CRO *Ceftriaxone, *CXM *Cefuroxime, *CAZ* Ceftazidime, *CIP *Ciprofloxacin, *CN *Gentamicin, *MEM *Meropenem, *TET *Tetracycline, *AN* Amikacin, *AZT* Aztreonam. The breaks (white line) in the graph indicate resistant isolates (where the bars are below the break) or sensitive isolates (bars extend beyond the break) to the respective antibiotics listed

All isolates were resistant to tetracycline, ampicillin, cefuroxime, ampicillin-sulbactam, sulfamethoxazole-trimethoprim and chloramphenicol because they did not show any in-vitro inhibition for *P. aeruginosa* isolates (zones of inhibition was ≤ 6 mm) (Fig. 3). EUCAST and CLSI have no guidelines for either zone of inhibition or MIC testing as *P. aeruginosa* is resistant to them (tetracycline, ampicillin, cefuroxime, ampicillin-sulbactam, sulfamethoxazole-trimethoprim, ceftriaxone and chloramphenicol). Both CLSI and EUCAST recommend that these antibiotics can be reported as resistant even without antibiotic testing. However for ceftriaxone, two *P. aeruginosa* isolates that were resistant to the other six antibiotics (tetracycline, ampicillin, cefuroxime, ampicillin-sulbactam, sulfamethoxazole-trimethoprim and chloramphenicol) exhibited very high zones of inhibition similar to the ATCC strains (Fig. 3). For interpretation of gentamicin, the CLSI breakpoint was used as EUCAST has no breakpoints (Fig. 3).

### Minimum inhibitory concentration (MIC) and minimum bactericidal concentration (MBC) assays

MIC assay was performed for ciprofloxacin against the isolates. MRSA isolates had MIC and MBC < 0.48 µg/mL. *E. coli* isolates had MIC and MBC ≤ 31.25 µg/mL whereas *P. aeruginosa* isolates was ≤ 1.95 µg/mL. *E. coli* isolates exhibited the highest MIC and MBC at 31.25 µg/mL for ciprofloxacin which was one-fold higher than the *E. coli* ATCC strain used (15.63 µg/mL) (Table 5). The ATCC strain for *S. aureus* had similar MIC and MBC as that of the MRSA isolates. M064 isolate of *P. aeruginosa* was two fold higher than it’s ATCC strain (Table 5).

**Table 5:** Minimum inhibitory concentration for MDR isolates and ATCC strains

	**Bacteria isolates**													
	***S. aureus***					***P. aeruginosa***						***E. coli***		
	**MRSA isolates**				**ATCC**	**PA isolates**					**ATCC**	**EC isolates**		**ATCC**
	**C015**	**C085**	**C091**	**C129**	**SA-25923**	**M032**	**M034**	**M064**	**M066(1)**	**M066(2)**	**PA-27853**	**M103**	**M165**	**EC-25922**
**MIC**	< 0.48	< 0.48	< 0.48	< 0.48	< 0.48	< 0.48	< 0.48	1.95	< 0.48	< 0.48	< 0.48	31.25	31.25	15.63
**MBC**	< 0.48	< 0.48	< 0.48	< 0.48	< 0.48	< 0.48	< 0.48	1.95	< 0.48	< 0.48	< 0.48	31.25	31.25	15.63

Key: *ATCC* American typed culture collection, *MRSA* Methicillin resistant, *PA P*. *aeruginosa*, *EC E. coli*, *MBC* Minimum bactericidal concentration, *MIC* Minimum inhibitory concentration, *All* concentrations in µg/mL

### Genotype distribution

**Fig. 4:**
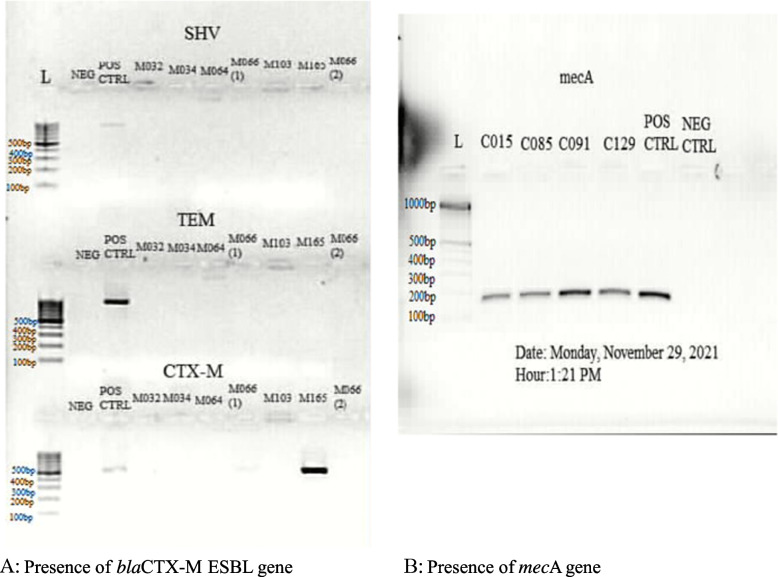
Presence of resistance genes in MDR

The *mec*A and ESBL (*bla*CTX-M, *bla*SHV and *bla*TEM) genes were amplified in the multi-drug resistant isolates using PCR (Table 6, Fig. 4A, B). The *bla*-CTX-M ESBL gene was found in one of the MDR *E. coli* isolates (Fig. 4A). However, no ESBL genes were detected in the MDR *P. aeruginosa* isolates. All MDR MRSA isolates were positive for the *mec*A gene (Fig. 4B).

**Table 6:** Forward and reverse primers used

Gene name	Primer name	Primer sequence
ESBL genes (*bla*SHV, *bla*TEM and *bla*CTX-M)	SHV-F	5'- GCC GGG TTA TTC … -3'
	SHV-R	5'- ATG CCG CCG CCA … -3'
	TEM-F	5'- GTA TCC GCT CAT … -3'
	TEM-R	5'- TCT AAA GTA TAT … -3'
	CTX-M-F	5'- TTT GCG ATG TGC … -3'
	CTX-M-R	5'- CGA TAT CGT TGG … -3'
*mec*A	*mec*A P4 2821	TCCAGATTACAACTTACCAGG
	*mec*A P7 2822	CCACTTCATATCTTGTAACG

## Discussion

Recent estimate puts the number of people infected with LF at 58 million, with majority of that population in developing countries. Long term exposure to LF results in damage of the lymphatics leading to lymphoedema and elephantiasis [37]. These result in mobility challenges, worsened financial status due to inability to engage in economic activities and put LF patients at risk of developing secondary infections due to ADLA [38]. In this study it is evident that majority of the participants reported with varied degrees of swelling and wounds that affect their ability to engage in economic activities. This also puts them at risk of bacterial infections (Table 1).

Previously, we have shown a positive correlation of the disease burden and pain or discomfort [39]. In managing the pain and skin peelings (as measures for morbidity management), LF patients resort to the use of antibiotics for self-medication as a preferred alternative to travelling long distance and the cost in accessing primary healthcare [5, 37]. Long exposure to antibiotics is known to be associated with resistance and this is supported by the fact that countries with higher antibiotic usage show higher resistance rates [40]. Antibiotic misuse due to self-medication has also resulted in the emergence of multi-drug resistance strains (including MRSA, cephalosporin-resistant *E. coli* and carbapenem resistant *P. aeruginosa*) with greater risk of infection and reduced therapeutic options [41, 42].

Bacterial infection in LF could worsen disease prognosis; hence knowledge on self medication and sensitivity patterns of co-infecting bacteria is essential in better management strategies (Tables  3, 4 and 5; Figs. 1, 2 and 3). Results indicated that participants have been self-medicating for a long time (evident in how easily they could assess and recall the antibiotics they used). Among the seven reported frequently used antibiotics by participants, the beta-lactam groups of antibiotics (amoxicillin, ampicillin, penicillin, flucloxacillin) represented 72% (n=36) followed by chloramphenicol (n=11, 22%), tetracycline (n=2, 4%) and clindamycin (n=1, 2%) (Table 2). This trend is similar to reports of amoxicillin, flucloxacillin, tetracycline and ampicillin being the commonest pharmacy antibiotic medicines used in Ghana [43]. In a similar report, amoxicillin is one of the most prescribed antibiotics in the United States of America [44].

Morbidity and mortality become more pronounced when polymicrobial bacterial infection are found in immunosuppressed individuals as in LF [6, 11]. Majority of participants presented with wounds that have been on them for ≥ 7 months with varied signs/symptoms of infections. Kathamuthu *et al.* [11] reported higher burdens of *Mycobacterium tuberculosis* in filarial infections as a result of immunomodulation (through upregulation of pathogenic chemokines and cytokines and downregulating key protective chemokines and cytokines of patients). Against the backdrop of the difficulty in curbing antimicrobial resistance, it was important to assess the phenotypic and genotypic characteristics of *S. aureus*, *E. coli* and *P. aeruginosa* isolates in LF patients. Among the Gram-negatives isolates, *E. coli* isolates were more resistant to the number of antibiotics tested compared to *P. aeruginosa* isolates. The results also showed that Gram-negative isolates from LF are beginning to exhibit higher MIC values when compared to typed strains that are not exposed to selective antibiotic pressure.

Persistent consumption and misuse of antibiotics enhance the acquisition and dissemination of drug resistant organisms (and genes) through environmental bacteria [40, 45–47]. This seems to be the phenomenon observed in this study; where the consumption of antibiotics (especially the beta-lactam antibiotcs) could have accounted for the development of MDR strains. In addition, easy access to antibiotics and lack of qualified personnel to dispense and counsel on appropriate antibiotic use in remote regions (study site) could account for the results obtained [43, 48].

*S. aureus* isolates exhibited the highest resistance to penicillin (88.46%) with erythromycin (7.69%) recording the lowest (Table 3). Similar reports have been observed in other studies because beta-lactam antibiotics continue to remain the mainstay of antibiotics treatment, hence the emergence of *mec*A and *blaZ* carrying MRSA strains [49–54]. Reported all isolates from chronic wounds with methicillin resistance to be positive for the *mec*A gene and resistant to other commonly used antibiotics (penicillin, tetracyline and trimethoprim/sulfamethoxazole). Aggarwal *et al*. [53] and Schaumburg *et al*. [54] have reported on the multi-drug resistant nature and high resistance of *S. aureus* for penicillin (97%), tetracycline (74%) and tremethiprim/sulfamethoxazole (77%).

Our findings show that all the MRSA (resistant to two or more additional antibiotics) were positive for the *mec*A gene as have been reported [51, 55]. MRSA percentage (15.38%) in this study is in line with the 15.15% reported in surgical wounds [56]. An earlier report by Wireko *et al*. (2021) [50] hints at a possible horizontal gene transfer for *mec*A in lymphatic filariasis MRSA isolates, owning to the presence of *mec*A gene is *Staphylococcus epidermidis*, *Staphylococcus haemolyticus* and *Staphylococcus hominis* isolates [50]. However, transposons have also been shown to be responsible for the translocation of resistance genes in the staphylococcal cassette chromosome, making MRSA resistant to other antibiotics [57].

All the *E. coli* isolates were resistant to tetracycline, ampicillin, sulbactam-ampicillin and sulfamethoxazole-trimethoprim and this could result in therapeutic failures (or higher concentrations will be needed) in morbidity management of LF patients (Table 3). In addition, more than half of the isolates were resistant to ciprofloxacin, ceftriaxone, cefuroxime and ceftazidime (Table 3). One MDR *E. coli* isolate was resistant to all the eleven [11] antibiotics tested with the exception of meropenem and paints a worrying sight of a possibility of extended drug-resistant strain (Fig. 2). Sensitivity tests for *P. aeruginosa* isolates were done but not reported for tetracycline, sulfamethoxazole-trimethoprim, chloramphenicol, ampicillin, ampicillin-sulbactam,ceftriaxone and cefuroxime as EUCAST and CLSI have no breakpoints for either the disk or MIC tests (Table 4). It was realized that the zones of inhibition for these antibiotics on MDR *P. aeruginosa* were ≤ 6 mm except ceftriaxone that had some isolates having zones > 15 mm (Fig. 3).

*P. aeruginosa* has been shown to acquire resistance under therapy and this has selected for multi-drug resistant strains [58–60]. The results agree with the selection of multi-drug resistant strains for *P. aeruginosa* as the isolates were found to be resistant to the antibiotics tested. The result (as shown with ceftriaxone) also corroborate reports by EUCAST and CLSI that some percentage of wild strains of *P. aeruginosa* are susceptible either due to a mutation or low levels of resistance [61, 62]. This further supports the antibiotic usage of the participants (none reported using any cephalosporin antibiotic) as selective pressure for cephalosporin resistance is low in this study.

*P. aeruginosa* has an innate resistance to the beta-lactam groups as such antipseudomonal penicillins in combination with beta-lactamase inhibitors is recommended [63]. In this study, it was evident that *P. aeruginosa* was not sensitive to ampicillin and ampicillin-sulbactam, which are part of the beta-lactam group. The observed of innate resistance of *P. aeruginosa* for ampicillin, ampicillin-sulbactam and **s**ulfamethoxazole-trimethoprim in this study has also been reported [64, 65]. Meropenem resistance occurred in 37.50% (*n* = 3) of *P. aeruginosa* isolates (40% in MDR isolates), which is similar to that reported by Bediako-Bowan and colleagues (Table 3; Fig. 3) [56].

Current evidence has shown that MDR bacteria have become prevalent, even in community-acquired infections [66, 67]. A total of 11 MDR were identified, representing 28.2% of bacteria isolated from the patients (Tables 4, 7, 8 and 9). 40.00% of isolates of *E. coli* were MDR and this is in line with the 33.33% that was reported by Nigussie *et al.* [65]. While Nigussie and colleagues reported 80.0% MDR *P. aeruginosa* isolates and 10.0% MRSA isolates, this study reports 62.5 and 15.38% as the proportions of MDR *P. aeruginosa* and MRSA isolates, respectively (Table 4) [65]. While findings from other diseases and niches have shaped the discourse of the genes driving these community infections, few have come from studies related to lymphatic filariasis [68].

**Table 7:** Phenotypic characterization of MDR methicillin resistant *S. aureus*

Isolates	Catalase	Coagulase	B-hemolytic
C091	Yes	Yes	Yes
C015	Yes	Yes	Yes
C085	Yes	Yes	Yes
C129	Yes	Yes	Yes

Key: Yes, means isolate is positive for a test

**Table 8:** Phenotypic characterization of MDR *E. coli* and *P. aeruginosa*

Isolates	LF/NLF	Oxidase	Indole	Citrate	TSI		
					Slant	Butt	Gas
M032	NLF	Yes	No	Yes	Red	Yellow	No
M034	NLF	Yes	No	Yes	Red	Orange red	No
M064	NLF	Yes	No	Yes	Red	Orange red	No
M066(1)	NLF	Yes	No	Yes	Red	Orange red	No
M066(2)	NLF	Yes	No	Yes	Red	Yellow	No
PA-27853	NLF	Yes	No	Yes	Red	Orange red	No
M103	LF	No	Yes	No	Yellow	Yellow	No
M165	LF	No	Yes	No	Yellow	Yellow	Yes
EC-25922	LF	No	Yes	No	Yellow	Yellow	Yes

Keys: *NLF* Non lactose fermenter, *LF* Lactose fermenter. Yes = Positive for that test

No = Negative for that test, M103 and M165 = *E. coli* isolates

M032, M034,M064, M066(1) and M066(2) = *P. aeruginosa* isolates

PA-27853 and EC-25922 = ATCC strains of *P. aeruginosa* and *E. coli* respectively

Red/Yellow (Slant/Butt) = Indication of dextrose fermentation only

Red/Orange red (Slant/Butt) = Absence of carbohydrate fermentation

Yellow/Yellow (Slant/Butt) = Indication of fermentation of dextrose, lactose and/or sucrose

**Table 9:** Zone of inhibition for MDR isolates against antibiotics

	Penicillin		Phenicol	Cephalosporin			Fluoroguinolone	Aminoglycoside	Carbapenem	Tetracycline	Sulfonamide	Aminoglycoside	Monobactam
**Gram-negative**	**AM**	**SAM**	**C**	**CRO**	**CXM**	**CAZ**	**CIP**	**GM**	**MEM**	**TET**	**SXT**	**AN**	**ATM**
**M032**	6.00	6.00	6.00	24.33	6.00	31.00	35.67	29.67	31.00	8.67	6.00	23.66	31.00
	+ 0.00	+ 0.00	+ 0.00	+ 0.33	+ 0.00	+ 0.57	+ 0.88	+ 0.88	+ 0.57	+ 0.33	+ 0.00	+ 0.33	+ 0.57
**M034**	6.00	6.00	6.00	25.33	6.00	28.67	36.00	22.67	20.33	8.33	6.00	24.66	20.33
	+ 0.00	+ 0.00	+ 0.00	+ 0.33	+ 0.00	+ 0.66	+ 0.57	+ 0.66	+ 0.33	+ 0.33	+ 0.00	+ 0.33	+ 0.33
**M064**	6.00	6.00	6.00	6.00	6.00	14.67	26.67	12.67	24.00	6.33	6.00	27.33	19.66
	+ 0.00	+ 0.00	+ 0.00	+ 0.00	+ 0.00	+ 0.88	+ 0.33	+ 1.85	+ 0.57	+ 0.33	+ 0.00	+ 1.45	+ 0.33
**M066(1)**	6.00	6.00	6.00	15.00	6.00	14.67	34.00	14.00	13.67	6.00	6.00	23.00	24.66
	+ 0.00	+ 0.00	+ 0.00	+ 0.57	+ 0.00	+ 0.66	+ 0.57	+ 0.57	+ 0.33	+ 0.00	+ 0.00	+ 1.15	+ 0.33
**M066(2)**	6.00	6.00	6.00	10.00	6.00	18.67	23.67	21.00	11.00	6.00	6.00	21.00	8.33
	+ 0.00	+ 0.00	+ 0.00	+ 0.57	+ 0.00	+ 0.33	+ 0.33	+ 0.57	+ 0.57	+ 0.00	+ 0.00	+ 0.57	+ 0.33
**PA-27853**	6.00	6.00	6.00	29.00	6.00	29.33	29.00	24.33	25.00	6.00	6.00	30.33	23.66
	+ 0.00	+ 0.00	+ 0.00	+ 0.57	+ 0.00	+ 0.66	+ 0.57	+ 0.66	+ 0.00	+ 0.00	+ 0.00	+ 0.33	+ 0.66
**M103**	6.00	10.33	6.00	10.00	6.00	16.33	6.00	8.00	27.00	6.00	6.00		
	+ 0.00	+ 0.33	+ 0.00	+ 0.57	+ 0.00	+ 0.33	+ 0.00	+ 0.57	+ 0.57	+ 0.00	+ 0.00		
**M165**	6.00	12.67	6.00	8.33	6.00	15.00	6.00	22.67	26.00	6.00	6.00		
	+ 0.00	+ 0.33	+ 0.00	+ 0.37	+ 0.00	+ 0.57	+ 0.00	+ 0.66	+ 0.57	+ 0.00	+ 0.00		
**EC-25922**	20.00	21.00	29.67	29.67	22.00	30.00	30.67	22.67	27.67	23.33	25.67		
	+ 0.00	+ 0.57	+ 1.45	+ 1.45	+ 0.00	+ 0.00	+ 0.66	+ 0.33	+ 0.66	+ 0.33	+ 0.33		
	Cephalosporin	Phenicol	Fluoroguinolone	Lincosamide	Macrolide	Aminoglycosides	Tetracycline	Penicillin	Sulfonamide				
**Gram-positive**	**FOX**	**C**	**CIP**	**CC**	**E**	**GM**	**TET**	**P**	**SXT**				
**C091**	16.00	6.00	28.33	26.00	26.67	22.67	6.00	10.33	27.33				
	+ 0.57	+ 0.00	+ 0.33	+ 0.57	+ 0.88	+ 0.33	+ 0.00	+ 0.33	+ 2.33				
**C015**	15.00	8.67	27.67	22.33	15.00	22.67	13.00	8.33	30.33				
	+ 0.57	+ 0.33	+ 0.33	+ 0.33	+ 0.57	+ 0.33	+ 0.57	+ 0.33	+ 0.88				
**C085**	15.67	22.67	23.67	24.00	26.00	21.67	9.00	11.00	28.33				
	+ 0.33	+ 0.66	+ 0.66	+ 0.57	+ 1.00	+ 0.66	+ 0.00	+ 0.00	+ 0.88				
**C129**	17.00	7.33	27.33	28.00	25.67	21.33	14.33	12.00	30.67				
	+ 0.00	+ 0.33	+ 0.33	+ 0.57	+ 0.88	+ 0.33	+ 0.33	+ 0.57	+ 0.88				
**SA-25923**	22.33	20.33	20.67	22.00	22.33	20.67	22.00	26.67	23.33				
	+ 0.33	+ 0.33	+ 0.66	+ 0.57	+ 1.20	+ 0.33	+ 1.52	+ 1.76	+ 1.45				

Key: PA-27853, SA-25923 and EC-25922 = American Type Culture Collection for *P. aeruginosa*, *S. aureus* and *E. coli*

Our findings showed the presence of *bla*CTX-M ESBL gene in one of the MDR *E. coli* isolates, while none of the tested ESBL genes were found in the *P. aeruginosa* isolates. The presence of* bla*CTX-M in *E. coli* may explain the resistance (antibiotic resistance profile) that was evident in the MDR isolates as this gene is involved in co-resistance with fluoroquinolones, extended activity against cefotaxime compared to ceftazidime and being predominant in community infections (Fig. 4B) [69, 70]. Furthermore, physiological activities such as low expression of porins (OmpC and OmpF in *E. coli*) that are transporters of antibiotics (quinolones, tetracycline, β-lactams or chloramphenicol) may also result in resistance due to low accumulation of antibiotics in bacteria [24]. Most studies including this, have focused on inheritable traits as the cause of antibiotic resistance. However, the absence of the ESBL genes in *P. aeruginosa* isolates points to the absence of genetic change from these group of genes, possibly due to bacterial permeability or other genetic factors [24].

The fact that the ciprofloxacin MIC of all the MDR isolates tested in this study falls within the sensitive range for each bacterium is welcoming considering the report by Newman and colleagues that puts the MIC for MDR *S. aureus* at > 0.256 mg/mL for ciprofloxacin. The ones obtained here had MIC < 0.00048 mg/mL (Table 5) [71]. All the MDR isolates in this study fell within the range given by EUCAST and Clinical & Laboratory Standard Institute (CLSI) as breakpoints [*P. aeruginosa* (0.001—0.5 mg/mL), *S. aureus* (0.001 – 1 mg/mL) and *E. coli* (0.25 – 0.5 mg/mL)] [61]. Comparing the ATCC strains with isolates from this study, we see a higher MIC value for some Gram-negative isolates. MDR isolates being sensitive to ciprofloxacin gives credence that these isolates have not been extensively subjected to selection pressure from some antibiotics tested in this study, as was indicated by the response from the antibiotic usage questionnaire. In essence, most inhabitants resort less to ciprofloxacin in treating their wounds and during filarial attacks.

This study has highlighted the phenotypic and genotypic (*mec*A, *bla*SHV, *bla*TEM and *bla*CTX-M) characteristics of MDR methicillin-resistant *S. aureus*, cephalosporin resistant *E. coli* and *P. aeruginosa* prevalence (Tables 1, 2, 3, 4, 5, 6, 7, 8 and 9; Figs. 1, 2, 3 and 4A, B). This is worrying, and it is expected that the findings of this study direct clinicians in the management of LF patients with secondary bacterial infections.

## Conclusion

This study reports the presence of MDR MRSA, cephalosporin resistant *E. coli* and carbapenem resistant *P. aeruginosa* in LF patients in the Ahanta West district of Ghana and the need for clinicians to be peculiar in how they administer antibiotics to these patients. In addition, this study has highlighted the antibiotic usage of participants and how it drives both phenotypic and genotypic modifications in the MDR isolates. These findings, especially the genetic mechanisms identified for the MDR resistance require further exploration such as sequencing to understand better the diversity occurring in these isolates and their impact on therapeutic failures in LF patients.

## Methods

### Study design and participants

Study participants were recruited from 8 LF endemic communities (Busia, Butre, Achowa, Princess Town, Akatakyi, Ampatano, Dixcove and Asemkow) in the Ahanta West Municipal. Ahanta West Municipal remains one of the hotspots in Ghana with a microfilariae prevalence of 2.2% and has been part of clinical trials to test doxycycline [72, 73]. This study followed all protocols regarding human participants and ethical clearance was given by the Committee of Human Research and Publications and Ethics, School of Medicinal Science, KNUST (CHRPE/AP/649/19). Participants who consented to the study included individuals with different stages of LF, who had lived in the endemic community for ten (10) years or more and were between the ages of eighteen (18) and sixty (60) years with wounds. These potential participants were enlisted by health extension workers prior to the study. Participants were given informed consent, which they consented to either by signing or thumb printing and countersigned by an independent witness.

### Sociodemography and wound characteristics

Sociodemographic data on age, occupation, marital status and sex were taken. Leg staging of participants were done as has been reported by [50]. Information of cause of wound and age of wound in months were done using a simple structured questionnaire. The size of wound, appearance of wound and signs of infection were reported by measuring and observing the wounds during the sample taking process.

### Data on antibiotic usage

To determine the possible impact of antibiotic usage on antimicrobial resistance in LF patients, a simple structured questionnaire was used to get their response as to whether they had used antibiotics within the last three months for treatment and, if any, to name the antibiotic.

### Collection of samples

Swabs were taken from LF wounds patients using Becton Dickson and Company (BBL) culture swab (plus amies gel medium without charcoal). Swabs were stored between 2—8 °C on the field, transferred to a liquid nitrogen tank (liquid nitrogen submersion) at the end of the day’s collection and transported to Kumasi Centre for Collaborative Research (KCCR) for culture and isolation of bacteria. The culture and isolation have been described previously [50]. *S. aureus* (ATCC 25923), *E. coli* (ATCC 25922) and *P. aeruginosa* (ATCC 27853) strains were used as controls and performed to specifications as is reported for sensitivity testing [74–76].

### Tests for identification of isolated organisms

#### Biochemical identification of MRSA

To identify *S. aureus*, coagulase test and catalase test were done on the Gram-positive isolates while observing for beta-hemolysis on blood agar (BA)*.* Organisms were cultured on BA or Mueller–Hinton agar (MHA) and incubated at 35 ± 2 °C for 18 ± 2 h. In determining catalase production in the isolates, colonies of bacteria from a fresh culture were picked from Mueller–Hinton agar and placed in droplets of catalase reagent (3% H_2_O_2_) and observed for bubble formation [77]. Bubble formation was indicative of a catalase positive isolate while no bubble formation indicated a negative test. The coagulase test was used to differentiate between *S. aureus* and coagulase-negative staphylococci. The coagulase test was performed by using fresh colonies from a culture. This was placed in a rabbit serum in a tube and incubated for 4 h, where the presence of clumping indicated a positive test, while no clumping indicated a negative test [78]. Beta-hemolysis was recorded as positive if there were formation of clear zones around the *S. aureus* isolates growth on BA that had been incubated at 35 ± 2 °C for 18 ± 2 h [78].

#### Biochemical identification of *P. aeruginosa* and *E. coli*

Gram-negative organisms were subjected to oxidase, indole, citrate and triple sugar iron (TSI) tests to aid in the identification and characterization of the phenotypes of *P. aeruginosa* and *E. coli* isolates. Bacteria isolates were cultured on MacConkey agar (MAC) or MHA and incubated at 35 ± 2 °C for 18 ± 2 h before all the tests were done. The oxidase test was performed to identify isolates that catalyze oxidase-reduction via the cytochrome C oxidase enzyme. To perform the oxidase test BBL Dryslide and Remel oxidase were used. Isolates grown on MHA were streaked on BBL Dryslide. For a positive test a bluish colouration was seen after 20 s, while no colour change indicated negative test [79]. This was confirmed by using the Remel oxidase reagent [80]. Indole production was used to differentiate Enterobacteria that degrade tryptophanase. Indole production test was done using 4–5 colonies of isolate grown on non-selective media (MHA) placed on a sterile swab stick [81]. The indole Remel reagent was then poured on the swab and within a period of 120 s, bluish colouration of the colonies indicated indole production (positive test), while pink colouration indicated no indole production (negative test). In determining bacteria isolates that possess citrate permease, the citrate utilization test was performed. Citrate utilization test employed the use of 5–6 colonies streaked on the slant of citrate agar. This was later incubated (35 ± 2 °C for 18 ± 2 h) and a bluish colouration was indicative of a positive test for citrate utilization [82]. TSI was done to differentiate Gram-negative enteric bacilli as previously described by [83].

### MALDI-TOF for confirmation of bacteria

Isolates of bacteria were grown on BA and later prepared for inoculation on the MALDI Biotyper [Bruker Daltonic GmbH] [50]. To confirm the isolates using ribosomal and housekeeping proteins, the MALDI-TOF was used. Generated spectra were compared with current libraries (MALDI Flex Control software system Server Version: 4.1.31 and 60 Hz Nitrogen Laser (337 nm wavelength) and log scores generated (Scores of the log were classified as: > 2.0 = High confidence identification, 1.7–2.0 = Low confidence and < 1.7.0 = Unreliable).

### Sensitivity testing and identification of multi-drug resistant MRSA, *E. coli* and *P. aeruginosa*

Having confirmed the isolates, it was important to assess the antimicrobial resistance of *S. aureus*, *P. aeruginosa* and *E. coli* in LF patients with wounds. Colonies of bacteria were picked from MAC/BA and inoculated in 1 mL of normal saline to give 0.5 McFarland standard. Cotton swab sticks (sterile) were dipped in the inoculum and spread on MHA plates that had passed quality control [61]. The plates were left to dry for 5 min and incubated at 35 ± 2 °C for 18 ± 2 h after appropriate antibiotic disks had been placed on the plates. The following antibiotics were used for *S. aureus*: cefoxitin (30 µg), erythromycin (15 µg), chloramphenicol (30 µg), tetracycline (30 µg), ciprofloxacin (5 µg), sulfamethoxazole-trimethoprim (23.75 µg /1.25 µg), clindamycin (2 µg), gentamicin (10 µg) and penicillin (10 UI). The following antibiotics were used for *P. aeruginosa*: ciprofloxacin (5 µg), ampicillin (10 µg), aztreonam (30 µg), sulfamethoxazole-trimethoprim (23.75 µg /1.250 µg), amikacin (30 µg), ampicillin-sulbactam (10 µg /10 µg), ceftriaxone (30 µg), gentamicin (10 µg), cefuroxime (30 µg), meropenem (10 µg), ceftazidime (30 µg) and tetracycline (30 µg). For *E. coli*, all the antibiotics used for *P. aeruginosa* were used with the exception of aztreonam (30 µg) and amikacin (30 µg). Zones of inhibition were read after incubation and breakpoints were recorded [61]. MDR *S. aureus*, *P. aeruginosa* and *E. coli* were identified as resistant to at least one antibiotic in three or more classes. The following antibiotic classes were used in this study: aminoglycosides, carbapenems, cephalosporins, fluoroquinolones, macrolides, monobactams, penicillins, sulfonamides, lincosamides, phenicols and tetracyclines.

### Minimum inhibitory concentration (MIC) assay

#### Preparation of inoculum

To gain insight into how the isolates from LF patients differed from typed strains (ATTC) in terms of inhibitory concentrations of antibiotics, MIC was performed using a broad-spectrum antibiotic (ciprofloxacin). Distinct colonies (5—6) grown on MHA were picked from an 18 ± 2 h culture and transferred to normal saline to give approximately 1.5 × 10^8^ cfu/mL (0.5 McFarland standard). A volume of 0.1 mL of 1.5 × 10^8^ cfu/mL inoculum was transferred to 9.99 mL of broth to give 1 × 10^6^ cfu/mL [84]. This was homogenized on a roller. An equal volume (100 µL) of an antimicrobial agent was added to the same volume of broth containing 1 × 10^6^ cfu/mL to give a final concentration of 5 × 10^5^ cfu/mL [85]. Tubes were inoculated within 30 min of standardizing the inoculum to maintain viable cell density.

#### Viable cell count and purity check

Viable cell counts were performed to ensure the final concentration of inoculum was 5 × 10^5^ cfu/mL. This was performed by taking 3 µL of the stock of inoculum (5 × 10^5^ cfu/mL) and diluting it in 3 mL of saline. A volume of 100 µL was taken and then spread on MHA plates, where an average of 50 colonies were counted from the final concentration. Purity plating was done by taking an inoculum and plating on an unselective medium (MHA) to check if growth is uniform throughout the plate.

#### Determination of MIC of ciprofloxacin using microdilution assay

Labelled wells of microtitre plates (1—11) were filled with 100 µL of broth (1 × 10^6^ cfu/mL), while well 12 was filled with broth without any bacteria inoculum. Test solutions of 100 µL were added to each well (1–8) in the microtitre plates to give concentrations in the range (128 mg/mL to 1 mg/mL). Wells 9 and 10 were filled with 100 µL of positive control (ciprofloxacin), while wells 11 and 12 were filled with normal saline [85]. This was then incubated at 35 ± 2 °C for 18 ± 2 h. To determine MIC, 20 µL of 3-(4,5-dimethylthiazol-2-yl)-2–5-diphenyltetrazolium bromide [MTT] (1.25 × 10^3^ mg/mL) was dispensed in each well and the sample re-incubated for 30 min [86]. MIC for ciprofloxacin against test organisms were indicated by the presence of a violet colouration (due to the production of insoluble formazan from MTT by viable cells). MIC was taken as the immediate well that did not show a violet colouration after 30 min of adding MTT.

### Minimum bactericidal concentration

To determine the concentration at which ciprofloxacin was bactericidal, an inoculum was taken from the MIC wells prior to the dispensing of MTT. The inoculum was plated on MHA plates and incubated at 35 ± 2 °C for 18 ± 2 h. The value of MBC was recorded after incubation.

### Phenotypic identification of MRSA and extended-spectrum beta-lactamases (ESBL)

MRSA was identified as *S. aureus* with a zone of inhibition < 22 mm for cefoxitin [30 µg] [61, 87]. Screening of suspected extended-spectrum beta-lactamases for *E. coli* and *P. aeruginosa* were done by plating the confirmed isolates on MacConkey plates with a concentration of 1 µg/mL of ceftazidime or cefotaxime. Isolates that grew on the ceftazidime or cefotaxime MAC agar underwent the double-disk synergy test [88].

### Extraction of DNA

ESBL-positive and MRSA isolates were examined for the presence of *bla*TEM, *bla*SHV, *bla*CTX-M and *mec*A using conventional PCR. DNA extractions were performed from overnight bacterial cultures using the boiling method [50, 89]. A loop full of bacteria was emulsified in 1 mL of phosphate-buffered saline and vortexed vigorously. The isolates were then centrifuged at 8000 rpm for 3 min and the supernatant discarded. A volume of 100 µL of nuclease-free water was added to the bacterial cells, vortexed vigorously and then incubated at 95 °C for 5 min. After incubation, centrifugation was done at 14,000 rpm for 5 min. The supernatant was pipetted into a well labelled 1.5 mL Eppendorf tube as DNA. The extracted DNA was stored at -20 °C until use. The extracted DNA were used as templates for the detection of the genes of interest.

### Amplification of gene

To determine possible genes potentiating resistance in the MDR isolates, PCR amplification was performed. Detection of *bla*TEM, *bla*SHV, *bla*CTX-M and *mec*A were performed using primers in Table 1. Amplification using the Veriti® thermal cycler was conducted using the following PCR conditions; an initial denaturation at 95.0 °C for 15 min; 35 cycles at 94 °C for 30 s, 60 °C for 1 min and 72.0 °C for 1 min. With a final extension of 72.0 °C for 7 min, the reaction was put on hold at 4 °C until ready to be viewed for bands. Each reaction volume was 25 µL and was done with modifications from Lin and colleagues protocol [90]. The following PCR conditions were used for *mec*A gene; an initial denaturation at 94.0 °C for 30 s; 30 cycles of 94 °C for 15 s, 45 °C for 1 min and 68.0 °C for 1 min. With a final extension of 68.0 °C for 5 min, the reaction was put on hold at 4 °C. Each reaction volume was 25 µL [91]. PCR products were visualized by agarose gel electrophoresis using UV-transilluminator (Vilber Lourmat, Collegien, France) after staining with ethidium bromide. The stained gel was captured onto a desktop computer using the infinity® software.

### Data analysis

Data were analyzed using Python (v 3.8.10), Matplotlib (v 3.3.3), Numpy (v 1.19.4) and Scipy (v 1.5.4).

## Supplementary Information

**Additional file 1.**

## Data Availability

The raw data supporting the conclusions of this article are included within this article.

## Abbreviations

ADLA: Acute dermatolymphangioadenitis
AMR: Antimicrobial resistance
ATCC: American Type Culture Collection
BA: Blood agar
CLSI: Clinical & Laboratory Standards Institute
ESBL: Extended-spectrum beta-lactamase
EUCAST: European Committee on Antimicrobial Susceptibility Testing
GPELF: Global Programme to Eliminate Lymphatic Filariasis
LF: Lymphatic filariasis
MAC: MacConkey agar
MALDI-TOF: Matrix-assisted laser desorption/ionization-time of flight
MDA: mass drug administration
MDR: multi-drug resistance
MHA: Mueller–Hinton agar
MMDP: morbidity management and disability prevention
MRSA: methicilin-resistant Staphylococcus aureus
TSI: Tripple Sugar Iron agar
WHO: World Health Organization
