# Correction: Multi-drug resistant bacteria isolates from lymphatic filariasis patients in the Ahanta West District, Ghana

**DOI:** 10.1186/s12866-022-02673-0

**Published:** 2022-11-03

**Authors:** Bill Clinton Aglomasa, Cynthia Kyerewaa Adu-Asiamah, Samuel Opoku Asiedu, Priscilla Kini, Emmanuel Kobla Atsu Amewu, Kennedy Gyau Boahen, Solomon Wireko, Isaac Kingsley Amponsah, Yaw Duah Boakye, Vivian Etsiapa Boamah, Alexander Kwarteng

**Affiliations:** 1 Department of Pharmaceutics, College of Health Science, Kwame Nkrumah University of Science and Technology, Kumasi, Ghana; 2 Kumasi Centre for Collaborative Research in Tropical Medicine, Kwame Nkrumah University of Science and Technology, Kumasi, Ghana; 3 Department of Clinical Microbiology, School of Medical Sciences, Kwame Nkrumah University of Science and Technology, Kumasi, Ghana; 4 Department of Biochemistry and Biotechnology, College of Science, Kwame Nkrumah University of Science and Technology, Kumasi, Ghana; 5 Department of Laboratory Technology, Kumasi Technical University, Kumasi, Ghana; 6 Department of Pharmacognosy, College of Health Science, Kwame Nkrumah University of Science and Technology, Kumasi, Ghana

**Correction: BMC Microbiol 22, 245 (2022)**.

10.1186/s12866-022-02624-9.

In the originally published version of this article four issues were noted. First, on page 1, second column, paragraph 1, line 16 reads, “**antibiotics and in are inhibited by beta-lactamase**…”. It should rather read, “antibiotics and are inhibited by beta-lactamase”. On the same page, column and paragraph, line 20 reads, “**blaSHV and blaCTX-M genes with the former being …**”. Instead it should read “*bla*SHV and *bla*CTX-M genes with the latter being …”. On page 8, column 1, paragraph 2, line 7 reads, “**[49–54]. Reported all isolates from chronic wounds with**”. Instead it should read “[49–54]. Wolters et al. reported all isolates from chronic wounds with “. “Wolters et al.“ was omitted. Lastly, on page 8, column 2, paragraph 6, line 7 reads, “**of innate resistance of P. aeruginosa for ampicillin, ampi-**“. Instead it should read “innate resistance of P. aeruginosa for ampicillin, ampi-“. The original article has been corrected.
