# Supplementary material for: Multi-drug resistant bacteria isolates from lymphatic filariasis patients in the Ahanta West District, Ghana
Source: BMC Microbiol. 2022 Oct 11;22:245. doi: 10.1186/s12866-022-02624-9 (PMC9552459; doi:10.1186/s12866-022-02624-9)
Supplement: Supplementary file 1 — Additional file 1. [file 12866_2022_2624_MOESM1_ESM.pdf]

Supplementary sheet for Fig 4

Fig 4: Presence of resistance genes in MDR

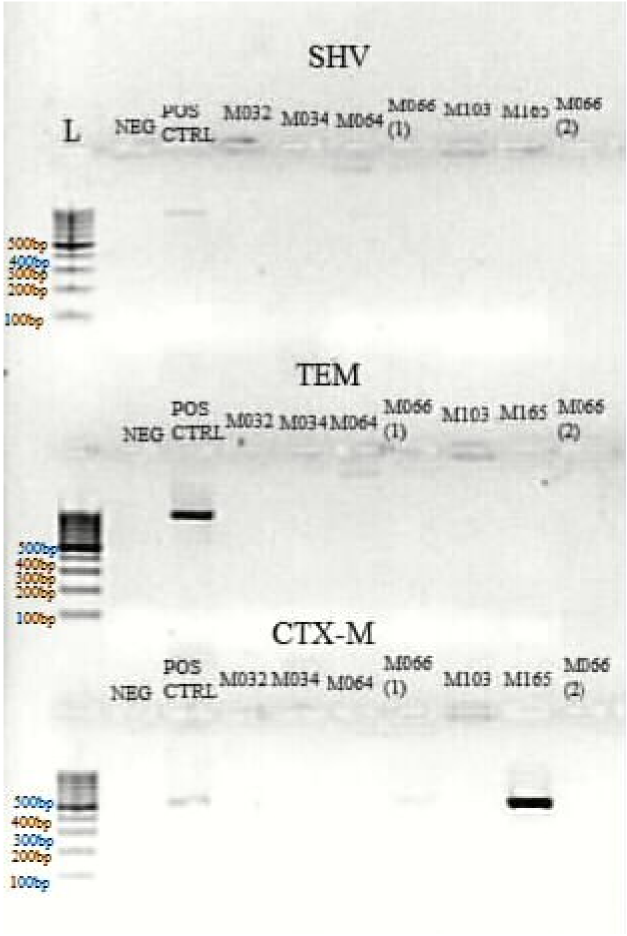

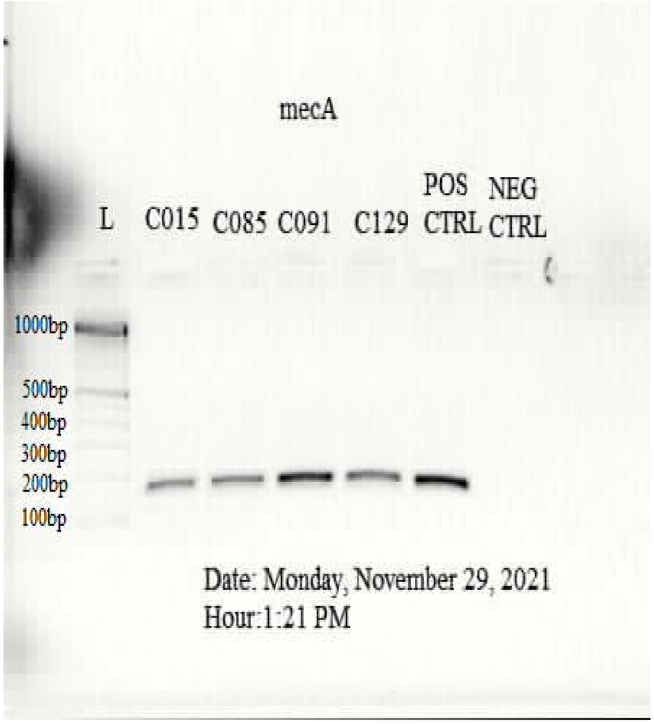

Fig 4A: Presence of *blaCTX-M* ESBL gene

Fig 4B: Presence of *mecA* gene

Fig 4A is the cropped image of the original gel. Fig4B is the original image of the gel. These images have their ladder sizes labelled.

Fig 4A: Presence of *blaCTX-M* ESBL gene

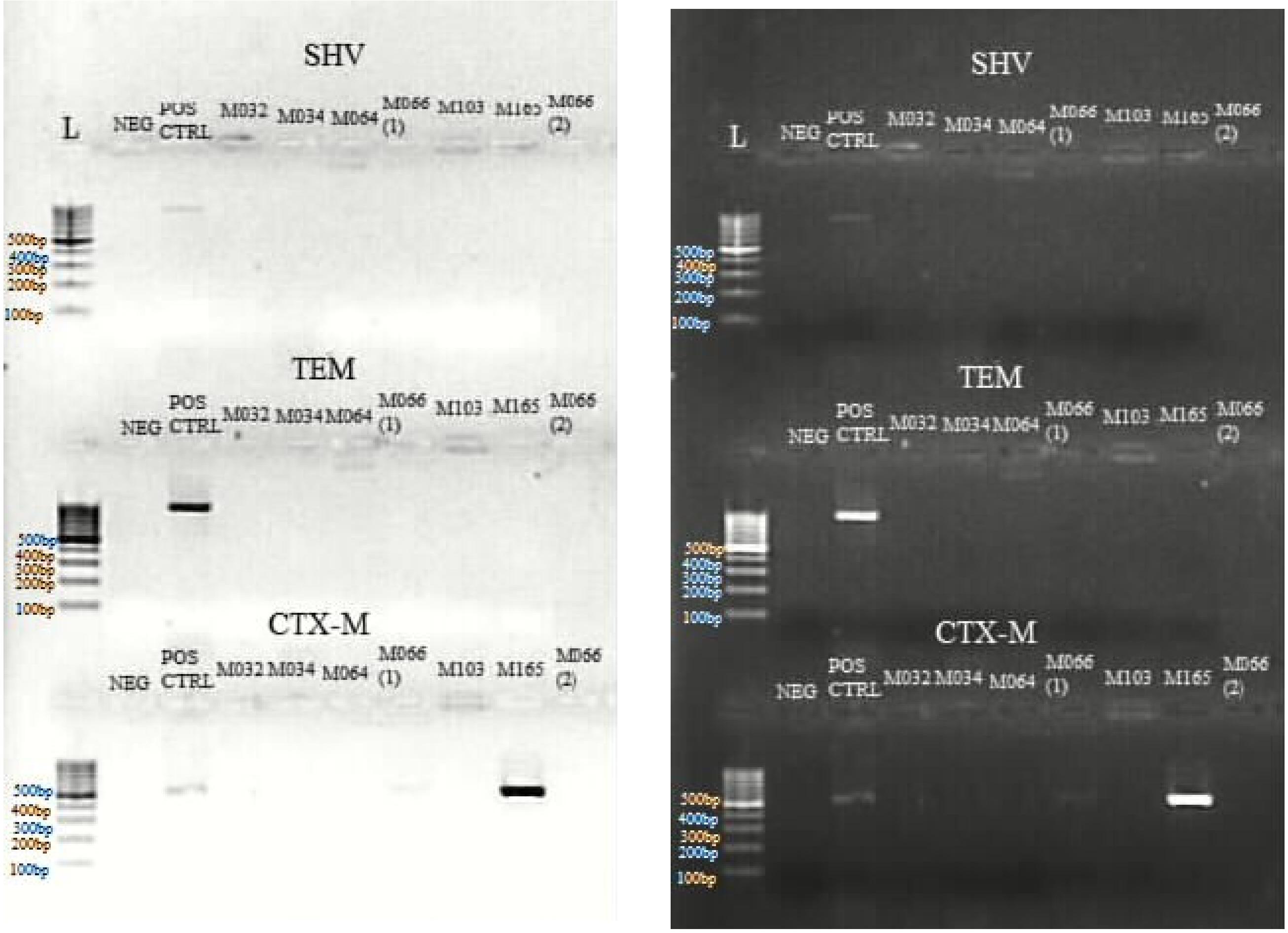

Fig 4A was cropped and different exposure and backgrounds were used to make the labelling more visible.

Fig 4B: Presence of *mecA* gene

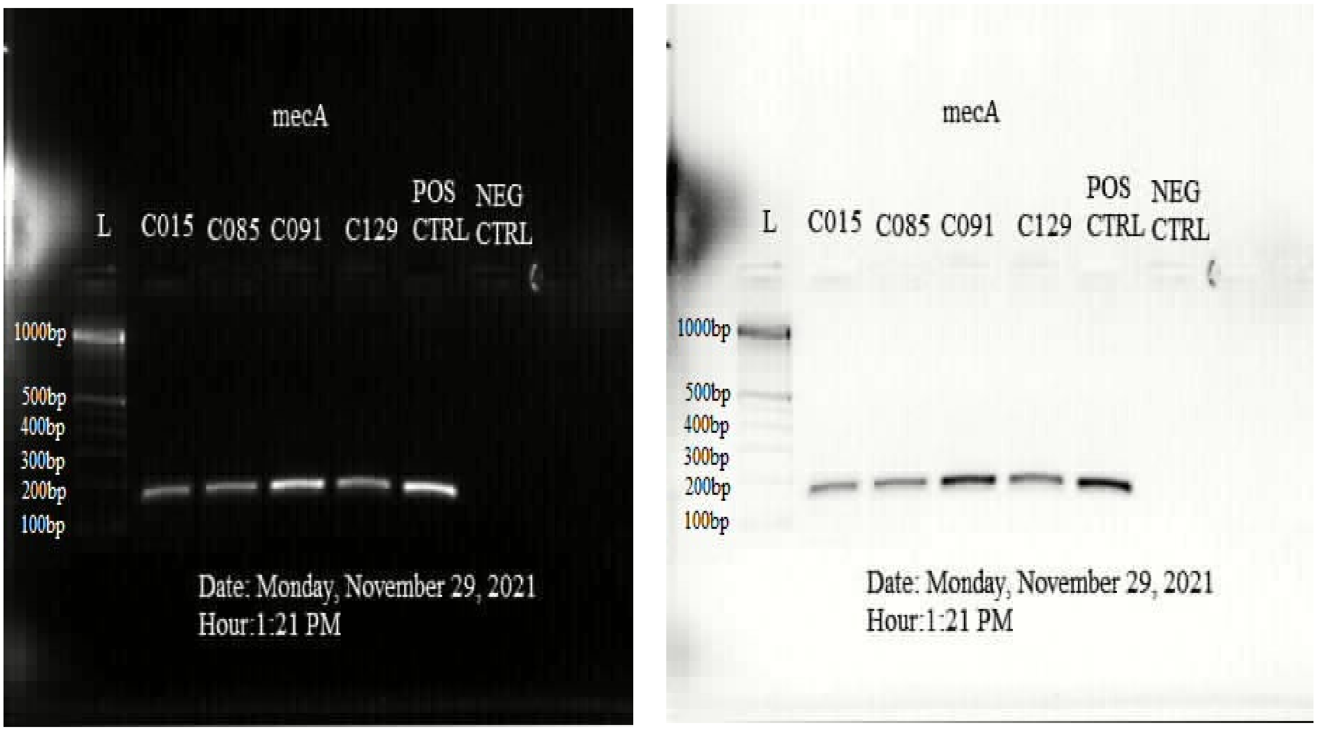

Different exposure and backgrounds were used to make the labelling of Fig 4B more visible.

Original images of the gels

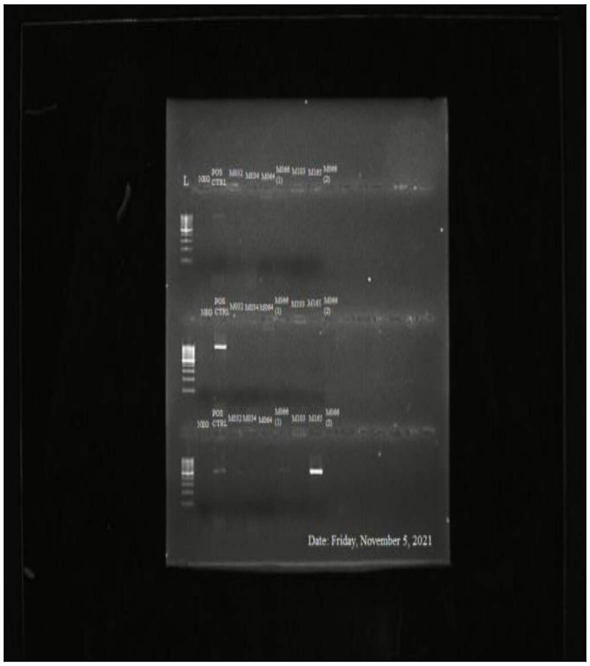

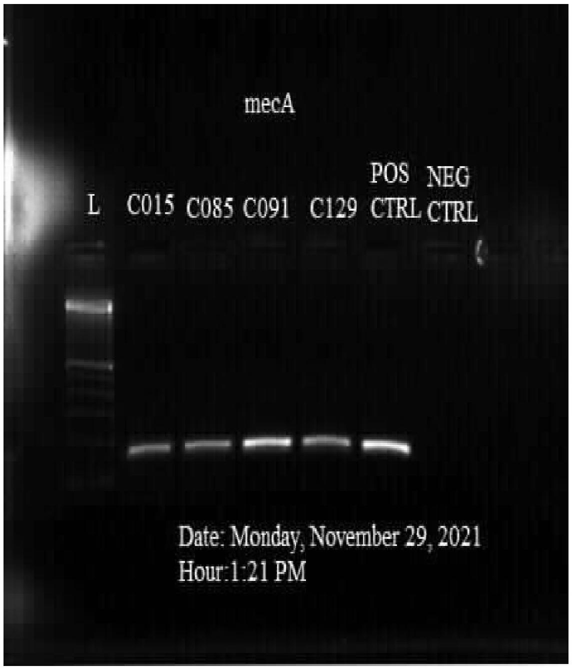

The gel for ESBL gene run

The gel for *mecA* gene run

The above are the original gels from the run.
